# An efficient method for the transduction of primary pediatric glioma neurospheres

**DOI:** 10.1016/j.mex.2018.02.006

**Published:** 2018-02-27

**Authors:** Michaël H. Meel, Dennis S. Metselaar, Piotr Waranecki, Gertjan J.L. Kaspers, Esther Hulleman

**Affiliations:** a Departments of Pediatric Oncology/Hematology, Neuro-oncology Research Group, Cancer Center Amsterdam, VU University Medical Center, De Boelelaan 1117, 1081HV, Amsterdam, The Netherlands; b Princess Máxima Center for Pediatric Oncology, Uppsalalaan 8, 3584CT, Utrecht, The Netherlands

**Keywords:** Serum-assisted lentiviral transduction, Pediatric Glioma, FBS, Neurospheres, Lentiviral transduction, Culture method, Diffuse Intrinsic Pontine Glioma

## Abstract

Pediatric high grade glioma (pHGG) and diffuse intrinsic pontine glioma (DIPG) are rare, but rapidly fatal malignancies of the central nervous system (CNS), and the leading cause of cancer-related death in children. Besides the scarcity of available biological material for research, the study of these diseases has been hampered by methodological problems. One of the major obstacles is the difficulty with which these cells can be genetically modified by conventional laboratory methods, such as lentiviral transduction. As a result, only very few successful stable modifications have been reported. As pHGG and DIPG cells are most often cultured as neurospheres, and therefore retain stem cell-like characteristics, we hypothesized that this culture method is also responsible for their resistance to transduction. We therefore developed a protocol in which pHGG and DIPG cells are temporarily forced to form an adherent monolayer by exposure to serum, to create a window-of-opportunity for lentiviral transduction. We here demonstrate that this protocol reliably and reproducibly introduces stable genetic modifications in primary DIPG and pHGG cells.

• Short-term serum exposure enables lentiviral transduction of primary pediatric glioma neurospheres.

## Graphical abstract

**Figure:**
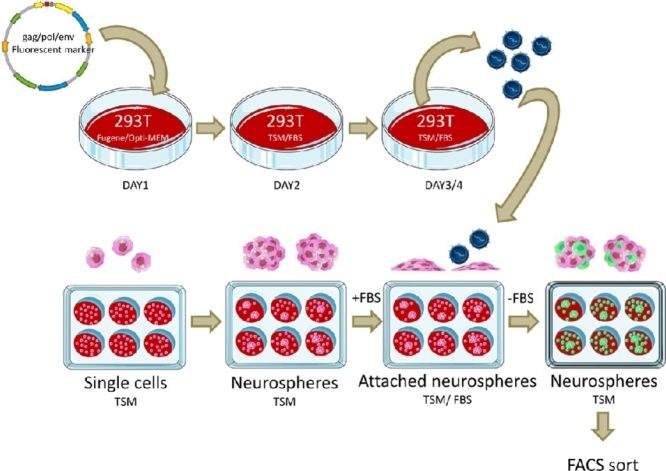

## Method details

### Background

Pediatric high grade gliomas (pHGG), including diffuse intrinsic pontine glioma (DIPG), are among the most lethal types of cancer occurring in children [[1], [2]]. Major advances in our understanding of the biology of the disease have been made in the past years. These advances include the discovery that these tumors are often driven by unique epigenetic events which are not seen in their adult counterparts, most importantly caused by mutations in the gene encoding Histone 3 [[3], [4]]. Besides being important for the development of a therapy for these diseases, the discovery of the unique epigenetic profile of pHGG and DIPG makes these tumors useful models for the study of epigenetic regulation of gene expression in general. However, research into these tumors and their epigenetic landscape has been hampered by the difficulty with which the tumor cells can be genetically modified. For unknown reasons, primary cultures of pHGG and DIPG cells are impervious to the introduction of genetic modifications by retro- or lentiviral transduction using standard laboratory techniques, which have been around since 1996 [5]. So far, only a few successful stable transductions of pHGG/DIPG cells have been reported [[6], [7], [8], [9]]. As primary pHGG and DIPG cells are generally cultured as neurospheres in serum-free medium, we hypothesized that this culture methodology is, at least partially, responsible for their resistance to retro- and lentiviral transduction, possibly as a result of the stem-like phenotype these cells adopt under serum-free conditions [[10], [11], [12]]. Alternatively, it is possible that fetal bovine serum (FBS) contains components that render cells susceptible to viral infection via unknown mechanisms. In line with this hypothesis, we successfully introduced genes in primary pHGG and DIPG cells by exposing these cells to FBS for a short period of time during the lentiviral transduction protocol. Hereby, we report the first reliable and reproducible lentiviral transduction protocol for primary pHGG and DIPG cells, allowing researchers to study their unique biological background and epigenetic landscape in more detail than before. This protocol has already been used to transduce primary DIPG neurospheres for use in a recent study by our group [13].

## Materials

### Reagents

DMEM/F12 with Phenol Red without glutamine (Thermo Fisher, Waltham, MA, USA, #12634010)

Neurobasal-A medium (Thermo Fisher, Waltham, MA, USA, #10888022)

Opti-MEM reduced serum medium (Thermo Fisher, Waltham, MA, USA, #31985070)

HEPES 1M (Thermo Fisher, Waltham, MA, USA, #15630056)

MEM Non-essential amino acid solution (Thermo Fisher, Waltham, MA, USA, #11140035)

GlutaMAX Supplement (Thermo Fisher, Waltham, MA, USA, #35050038)

Sodium Pyruvate 100 mM (Thermo Fisher, Waltham, MA, USA, #11360039)

B27 Supplement without vitamin A (Thermo Fisher, Waltham, MA, USA, #12587-010)

Basic Fibroblast Growth Factor (Peprotech, London, UK, #100-18B)

Epidermal Growth Factor (Peprotech, London, UK, #AF-100-15)

Platelet-derived Growth Factor AA (Peprotech, London, UK, #100-13A)

Platelet-derived Growth Factor BB (Peprotech, London, UK, #100-14B)

Heparin 5000 IE/mL (Local Pharmacy)

Primocin (Invivogen, San Diego, CA, USA, #ant-pm-2)

FuGENE HD (Promega, Madison, WI, USA, #E2311)

Accutase – Enzyme Cell Detachment Medium (Thermo Fisher, Waltham, MA, USA, #00-4555-56)

Fetal Bovine Serum (HyClone UK, Northumberland, UK, Cat #SV30180.03, Lot #SXM20001)

Plasmids for lentivirus production:

pMD2.G (https://www.addgene.org/12259/)

pRRE (https://www.addgene.org/12251/)

pRSV (https://www.addgene.org/12253/)

Lentiviral expression vector encoding gene of interest and fluorescent protein (in our example pSLIEW [14])

### Recipes

TSM Base:

250 mL Neurobasal-A medium

250 mL DMEM/F12 without Glutamax

5 mL HEPES 1M

5 mL MEM-Non-essential Amino Acid solution 100×

5 mL GlutaMAX Supplement 100×

5 mL Sodium Pyruvate 100 mM

1 mL Primocin

Store for up to 1 year at 4 °C. If using medium without antibiotics, store for 1 month maximum.

Complete TSM*

49 mL TSM Base

1 mL B27 supplement without vitamin A

20 ng/mL bFGF**

20 ng/mL EGF**

10 ng/mL PDGF-AA**

10 ng/mL PDGF-BB**

5 IE/mL Heparin**

*: Complete TSM can be stored for only 24 h at 4 °C due to quick degradation of growth factors in these solutions (especially bFGF).

**: stock solutions are more stable at high concentrations (∼1 mg/mL), with carrier proteins such as BSA, see manufacturers protocols for more details; also make aliquots as growth factors are sensitive to freeze/thaw cycles.

### Equipment

Axiovert 200 M Fluorescence microscope (Zeiss, Oberkochen, Germany), equipped with a PCO

Sensicam (PCO AG, Kelheim, Germany) and a Lambda DG-4 illumination system (Sutter Instrument, Novato, CA, USA)

Slidebook 6 Digital Microscopy Software (Intelligent Imaging, Göttingen, Germany)

Pre-Separation Filters (30 μm) (Miltenyi Biotec, Teterow, Germany, # 130-041-407)

BD FACSAria™ II Flow cytometer equipped with BD FACSDiva software v8.0.1 (BD Biosciences, MD, USA)

## Procedure

*Virus production – day 0*

Step 1: Heat-inactivate FBS by thawing it fully at room temperature, subsequently heating it at 56 °C for 30 min, then cooling it to 4 °C for short-term storage (maximum 2 weeks).

Step 2: Plate HEK293T at a density of 3*10^6^ cells per dish in a 100 mm cell culture dish in 17 mL TSM Base supplemented with 10% heat-inactivated FBS. Culture the cells in a cell incubator at 37 °C and 5% CO_2_ throughout the protocol.

*Virus production – day 1*

Step 3: Refresh medium of HEK293T cells four hours before transfection.

Step 4: Dilute a total of 19 μg of plasmid DNA (4,5 μg each of pMD2.G, pRRE, pRSV and plasmid of choice (here: pSLIEW) in 888 μL Opti-MEM.

NOTE: pSLIEW is the example plasmid used in the current protocol. Any plasmid that is suitable for lentivirus production and expresses a fluorescent selection marker can be used.

Step 5: Add 47 μL FuGENE HD to the Opti-MEM containing the plasmid DNA and mix well by pipetting gently up and down 10–15 times.

Step 6: Incubate the Opti-MEM/DNA/FuGENE HD mixture for 5 min at room temperature.

Step 7: Add 850 μL of the Opti-MEM/DNA/FuGENE HD mixture per dish to the HEK293T cells. Mix gently by rotating the culture dish several times.

*Virus production – day 2*

Step 8: After 24 h incubation, remove all medium with the transfection reagents from the HEK293T cells and add 17 mL fresh TSM Base supplemented with 10% heat-inactivated FBS to each dish.

*Virus production – day 3*

Step 9: Remove all medium from the HEK293T cells, store it at 4 °C and add 17 mL fresh TSM Base supplemented with 10% heat-inactivated FBS to each dish.

*Virus production – day 4*

Step 10: Remove all medium from the HEK293T cells and pool it with the medium harvested the previous day. Discard the culture dishes with the HEK293T cells.

Step 11: Filter the virus-containing medium through a sterile 0.45 μm filter. Filtered virus-containing medium can be used directly for downstream applications or stored at −80 °C for a maximum of 6 months.

*Lentiviral transduction of primary pediatric glioma cells – day 0*

Step 12: Dissociate neurospheres of primary cultures of pediatric glioma cells cultured in complete TSM *without* FBS.

Step 13: Seed glioma cells in 6-well plates at a density of 10^5^ cells/well in 1 mL complete TSM *without* FBS and wait for 24 h to allow for formation of small neurospheres. Culture the cells in a cell incubator at 37 °C and 5% CO_2_ throughout the protocol.

*Lentiviral transduction of primary pediatric glioma cells – day 1*

Step 14: Add 1 mL TSM Base supplemented with 10% heat-inactivated FBS to each well containing glioma neurospheres, and wait for 24 h to allow for (partial) attachment of glioma cells.

*Lentiviral transduction of primary pediatric glioma cells – day 2*

Step 15: Add 2 mL filtered virus-containing medium, either fresh or thawn, to each well containing (partially) attached glioma cells. Incubate cells with virus-containing medium for 48 h.

CAUTION: Not all primary pediatric HGG or DIPG cultures will form an adherent monolayer; some will form loosely adherent cell aggregates. However, some attachment and morphological changes of the cells need to be present. If not, wait up to 48 additional hours before adding virus medium. See troubleshooting section.

*Lentiviral transduction of primary pediatric glioma cells – day 4*

Step 16: Remove all medium from the cell cultures and wash each well carefully with 5 mL of PBS at room temperature.

CAUTION: If any neurospheres are still present in suspension, collect these by centrifugation of the removed medium at 250×*g* for 5 min at room temperature, then pool them with the remainder of the cells of step 17.

Step 17: Add 1 mL of Accutase to each well of the 6-well plate containing glioma cells, incubate the plate for 5 min at 37 °C, then collect cells by flushing the wells with 5 mL of PBS.

Step 18: Centrifuge collected cells for 5 min at 250×*g* at room temperature, remove supernatant and resuspend cells in 4 mL complete TSM *without* FBS. Allow cells to recover and form neurospheres for 5 days.

**Fig. 1:**
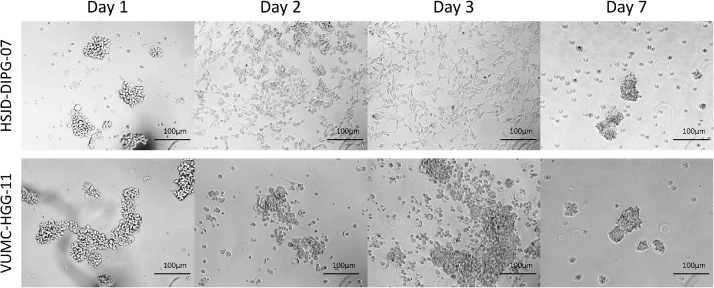
Response of primary DIPG and pediatric HGG neurospheres to 72 h of exposure to FBS (Day 1–3), with full recovery of neurosphere morphology within five days after withdrawal of serum exposure.

NOTE: The crucial step in this protocol is the exposure of the primary neurospheres cultures to FBS. After 24 h, most neurosphere cultures will start to form adherent monolayers, as is the case for the HSJD-DIPG-07 cells (Fig. 1). However, some primary neurospheres cultures will not adopt this typical morphology, instead forming loosely adherent aggregates of cells at the bottom of the culture wells, as is the case for the VUMC-HGG-11 cells (Fig. 1). In our experience, this does not influence transduction efficiency or cell survival throughout the protocol. Regardless of response, as soon as serum exposure has ended and culture medium has been changed to complete TSM, cells will revert to their original neurosphere morphology within 14 days.

*Lentiviral transduction of primary pediatric glioma cells – day 9*

Step 19: Evaluate transduction efficiency by fluorescence microscopy; proceed with step 20 only if any fluorescent cells are present.

NOTE: If no fluorescent cells are present, discard the cells and repeat the protocol from step 1. See troubleshooting section for adjustments that can be made to the protocol.

*Purification of successfully transduced primary pediatric glioma cells by FACS – day 1*

Step 20: Collect transduced cells from the culture flasks and centrifuge them at 250×*g* for 5 min at room temperature, then remove all culture medium from the cell pellet.

Step 21: Resuspend each cell pellet in 1 mL of Accutase and incubate the tubes for 5 min at 37 °C, then pipette cells up and down through a 1 mL pipette tip until a homogenous cell suspension is visible and add 9 mL of PBS to each tube and centrifuge the tubes at 250×*g* for 5 min at room temperature.

Step 22: Remove PBS with Accutase from the cell pellets and resuspend the pellets in 1 mL PBS. Then filter cells through a 30 μm pre-separation filter.

Step 23: Analyze expression of fluorescent proteins of transduced cells by fluorescence-activated cell sorting (FACS) using a flow cytometer capable of cell sorting and equipped with the appropriate lasers and filters. Include wild-type (WT) cells from the parental cell culture as a negative control to identify the threshold of fluorescent intensity for sorting.

NOTE: In our case, cells were sorted on a BD FACSARIA II flow cytometer based on their EGFP expression (excitation: 488 nm, emission filter 530/30 nm). However, as long as the appropriate lasers and filters are present, any flow cytometer with sorting module should suffice.

Step 24: Select cells with fluorescent intensity above the level of the negative control in the appropriate channel. Sort these cells using a 100 μm nozzle at a pressure of <25 PSI to minimize cell stress.

CAUTION: Although a nozzle with a smaller diameter or a higher sorting pressure may be used, this can increase cell stress and subsequent cell death.

HINT: Sorting efficiency can sometimes be improved by applying stringent thresholds for event identification on parameters such as forward scatter (FSC) or the fluorescent intensity in the channel of the fluorescent protein. Do not apply these thresholds during analysis, but only during sorting.

Step 25: Culture FACS-isolated cells in complete TSM *without* FBS. A cell concentration of 10^5^/mL is recommended. Allow cells to recover and form neurospheres for 7 days.

*Purification of successfully transduced primary pediatric glioma cells by FACS – day 8*

Step 26: Evaluate the purity of the sorted cells by fluorescence microscopy; if non-fluorescent cells are present, repeat steps 20–25.

NOTE: On some occasions a third cycle of cell sorting is necessary to achieve 100% purity of transduced cells. Perform this third cycle at least a week after the second round of purification.

## Method validation

**Fig. 2:**
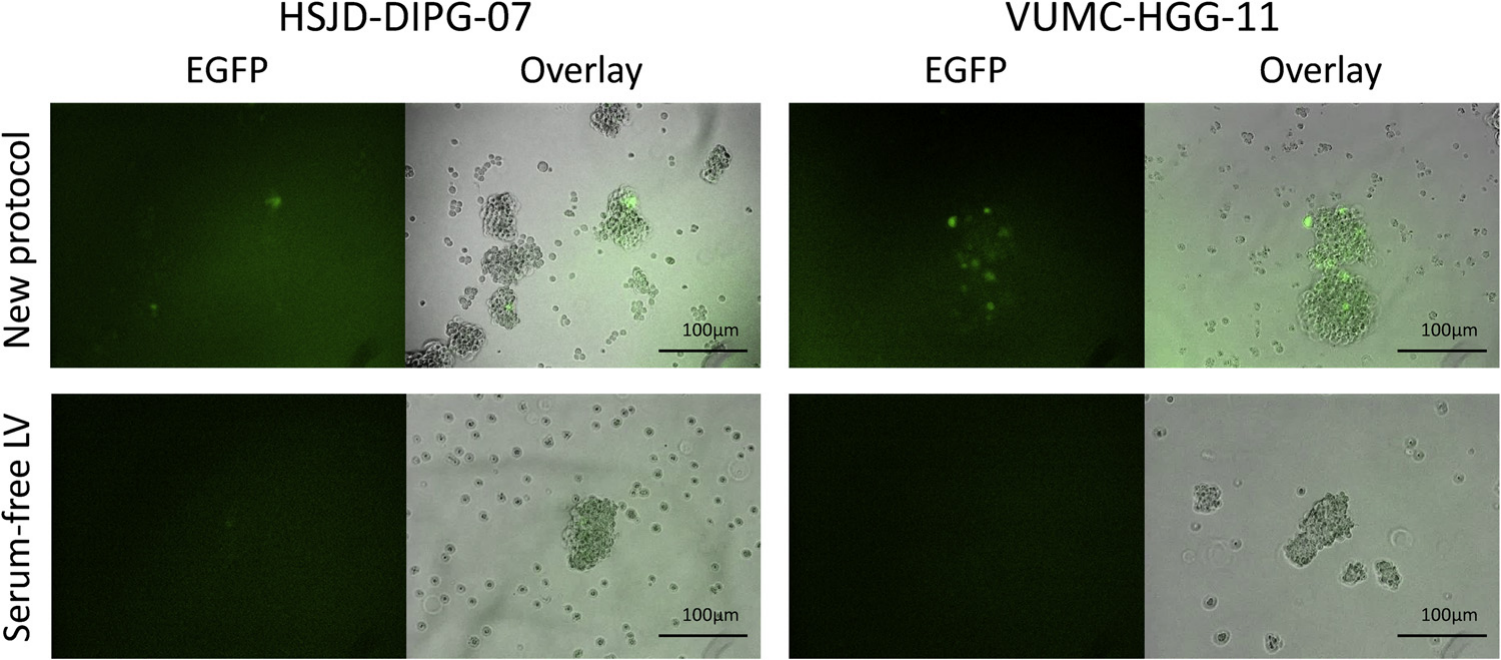
Fluorescence microscopy of DIPG and pediatric HGG neurospheres seven days after lentiviral transduction. Shown are overlays of fluorescence images on bright-field images (100× magnification). Upper panel shows neurospheres transduced by the described method, lower panel shows neurospheres exposed to lentivirus in serum-free conditions using a standard transduction protocol.

**Fig. 3:**
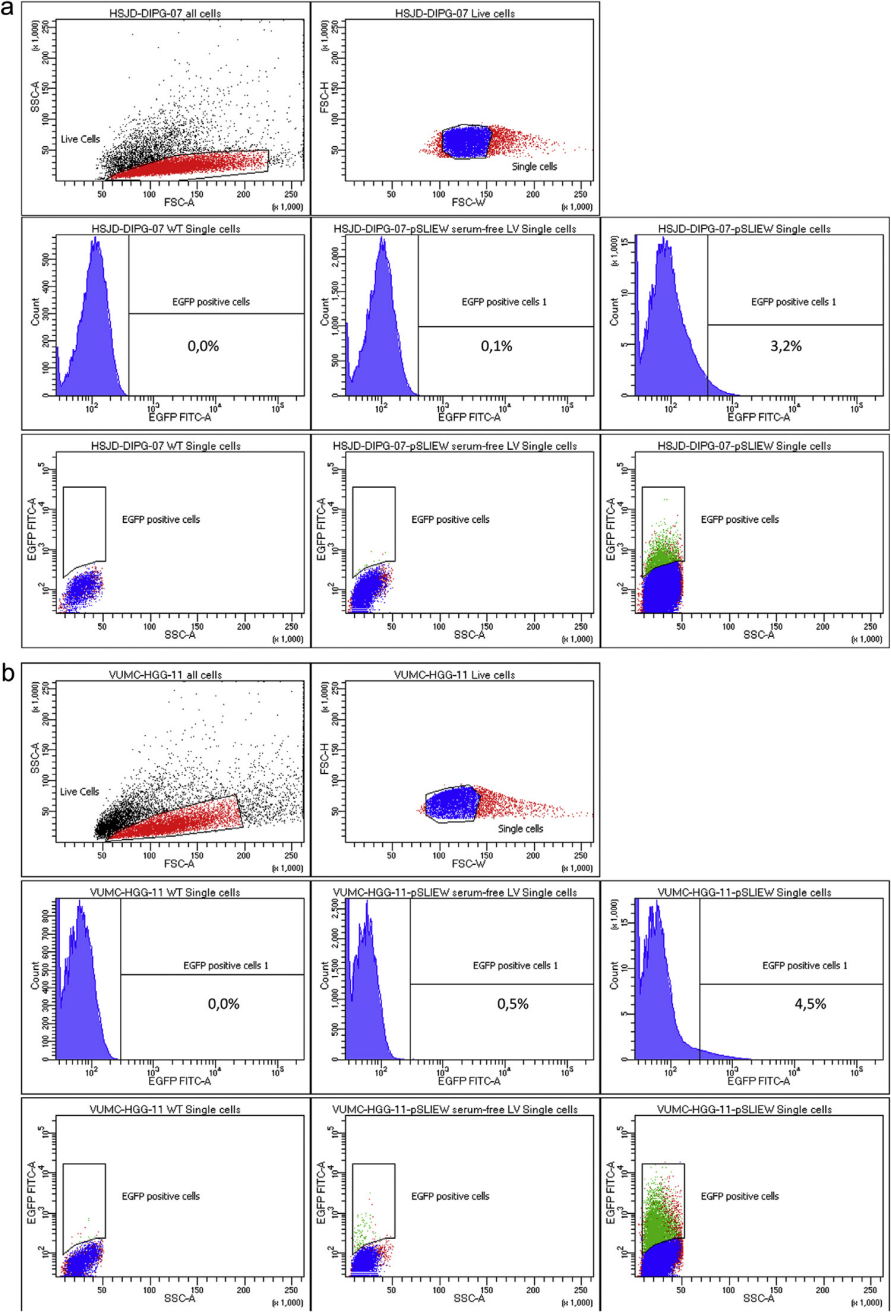
FACS analysis of primary DIPG and pediatric HGG cultures 7 days after lentiviral transduction. Top two panels of both (a) and (b) show gating strategy for selection of live HSJD-DIPG-07 and VUMC-HGG-11 cells respectively. Bottom six panels of both (a) and (b) show a comparison in EGFP fluorescence between WT cells (left two plots), cells transduced using a conventional serum-free lentiviral transduction protocol (middle two panels) and cells transduced using the described protocol (right two panels).

**Fig. 4:**
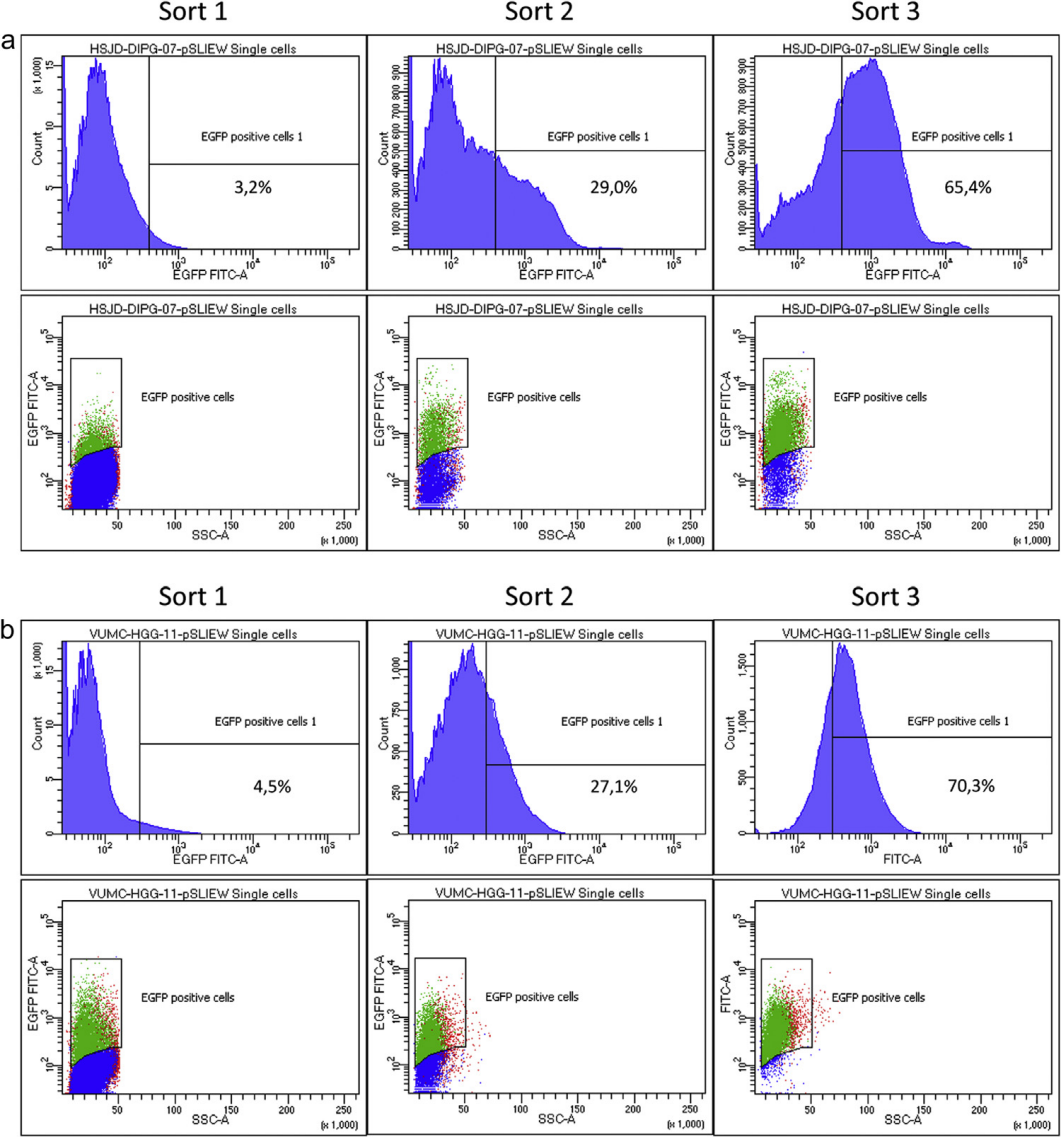
FACS analysis of HSJD-DIPG-07 (a) and VUMC-HGG-11 (b) cells subjected to three rounds of sorting to increase purity of transduced cells.

Primary pHGG and DIPG cultures are easily transducable using lentiviruses in the described protocol. However, to generate a culture in which all cells possess the genetic modification, selection procedures are needed. We have used, and recommend, fluorescent proteins as selection markers. Transduced primary pHGG/DIPG cells will start expressing fluorescent proteins several days after transduction; maximal expression is generally seen after 4–5 days. Using this protocol, expression of fluorescent proteins is observed in 1–10% of cells. In comparison, when using a standard lentiviral transduction protocol under serum-free culture conditions, less than 1% of the cells express fluorescent proteins; in many cases no successfully transduced cells are observed at all (Fig. 2). At the timepoint when maximal expression of fluorescent proteins is seen by fluorescence microscopy, transduced cells can be isolated by FACS. Expression levels of genes introduced by lentiviral transduction in primary pediatric HGG or DIPG cells are often relatively low. Therefore, a clear distinction between transduced and non-transduced cells cannot always be made by FACS (Fig. 3, lower right panels in a and b). In this case, the culture can be enriched for cells expressing fluorescent proteins by isolating all cells with fluorescence intensity above the background level of non-transduced cells. However, cells isolated this way will rarely consist for 100% of successfully transduced cells. Therefore, we recommend repeating the FACS sorting procedure at least once, a week after the first enrichment of cells expressing the fluorescent protein. This creates a stepwise increment in the percentage of successfully and stably transduced cells in the culture, and allows for the generation of a >99% pure culture (Fig. 4).

As prolonged exposure to FBS can induce differentiation of stem-like cells, concerns may exist with regard to the possibility that the described protocol induces permanent changes in the glioma cultures, reducing their relevance as a disease model. However, the short timeframe within which pHGG/DIPG cells are exposed to serum, and the rapid return to serum-free conditions seem to prevent permanent changes from occurring. The strongest evidence for this is the fact that cells transduced in this fashion retain their engraftment capacity; the hallmark of cancer stem cells. As an illustration; untransduced HSJD-DIPG-07 cells, as well as their counterparts transduced by the described method, display strongly resembling engraftment and survival of tumor-bearing mice in two recently published studies [[15], [16]]. Furthermore, a recent study by our group has shown that even prolonged exposure to FBS does not result in a reduction in expression of stem cell markers in DIPG cultures, making it even more unlikely that the short exposure used in this protocol has any influence on the stem cell characteristics of the transduced cells [17]. Nonetheless, we advise researchers using the described transduction method to validate the cell line identity, expression of stem cell markers and engraftment capacity of pHGG/DIPG neurospheres after transduction, as should be done after any lentiviral transduction procedure. Where applicable, researchers should also compare results of their studies between transduced and untransduced pHGG/DIPG cultures.

## Troubleshooting

Table 1 presents an overview of the most commonly encountered problems during transduction of primary HGG/DIPG cells using the described protocol. Virus production in HEK293T cells according to our protocol should not produce any difficulties, as the only difference with standard protocols is the use of TSM with FBS instead of DMEM-based culture media, a modification that is well tolerated by the HEK293T cells. Most problems arise from differences between primary cell cultures in their adaptation to serum-containing medium and the subsequent adaptation to serum-free medium. These problems can generally be solved by making slight changes in the concentration of FBS and/or the exposure time to serum-containing TSM. If transduction fails despite making adequate changes to these conditions, it is possible that the cells are unable to start transcription from the promotor of the gene of interest. In those cases, changes will have to be made to the plasmid containing the gene of interest. It is recommendable to design a plasmid in such a way that expression of fluorescent protein is directly coupled to the gene of interest, e.g. by gene fusion or connection of mRNA of the fluorescent protein to the gene of interest by use of an internal ribosomal entry site (IRES) or T2A linker sequence.

**Table 1:** Overview of troubleshooting per step of the protocol.

Step	Problem	(Probable) cause(s)	Suggestions
13	Delayed formation of neurospheres	- Cell stress induced by dissociation	1. Shorter incubation time of cells in Accutase 2. Wait an additional 24 h before proceeding with the protocol
14	Delayed attachment of glioma cells (no morphological changes observed)	- Cell stress induced by FBS exposure - Reduced response to FBS exposure	1. Adjust FBS% of TSM, final concentrations between 5 and 15% may be appropriate 2. Increase exposure time of glioma cells to FBS before addition of virus-containing medium; exposure times of up to 72 h may be needed
14	Excessive cell death after exposure to FBS	- High sensitivity of cultured cells to serum components	1. Reduce final concentration of FBS to a maximum of 5% 2. If reduction of FBS concentration does not prevent cell death, start with higher cell numbers (up to 5*10^5^/mL)
17	No detachment of cells after incubation with Accutase	- Strong adherence of cells to plastic after exposure to FBS	1. Increase incubation time of cells with Accutase to a maximum of 15 min 2. Decrease final concentration of FBS throughout the protocol to 5% 3. If both suggestions still result in a lack of detachment, gently scrape cells from the culture flask/well mechanically
18	Cells continue to form an adherent monolayer despite withdrawal of FBS	- Incomplete enzymatic digestion by Accutase of cell surface proteins involved in adherence - Induction of (partial) differentiation of tumor cells	1. Repeat step 16–18 2. Decrease final concentration of FBS throughout the protocol to 5% 3. Shorten incubation time in FBS-containing medium (eg 12 h before addition of virus medium and 24 h incubation with virus-containing medium)
19	No cells expressing fluorescent proteins	- Unsuccessful transduction - No transcription from integrated construct	1. Increase exposure time of glioma cells to FBS before addition of virus-containing medium; exposure times of up to 72 h may be needed 2. Increase exposure time of glioma cells to virus-containing medium; exposure times of up to 7 days are tolerated 3. Use adjuvantia that increase lentiviral transduction efficiency, such as Polybrene, during exposure to virus-containing medium 4. Change promotor of the gene encoding the fluorescent protein. CMV promotors tend to be poorly transcribed in primary glioma cells.
26	Cells lose expression of fluorescent proteins	- No stable integration of construct in host DNA - Epigenetic silencing of integrated construct	1. Repeat step 20–25 to enrich for stably transduced cells 2. Change promotor of the gene encoding the fluorescent protein. CMV promotors tend to be poorly transcribed in primary glioma cells.
